# A Compartmentalized Neuronal Cell-Culture Platform Compatible With Cryo-Fixation by High-Pressure Freezing for Ultrastructural Imaging

**DOI:** 10.3389/fnins.2021.726763

**Published:** 2021-09-08

**Authors:** Hung Tri Tran, Miriam S. Lucas, Takashi Ishikawa, Sarah H. Shahmoradian, Celestino Padeste

**Affiliations:** ^1^Laboratory of Nanoscale Biology, Paul Scherrer Institute, Villigen, Switzerland; ^2^Scientific Center for Optical and Electron Microscopy ScopeM, ETH Zürich, Zurich, Switzerland

**Keywords:** high pressure freezing, neuronal co-culture, focused ion beam – scanning electron microscopy, serial sectioning and imaging, photolithography, transmission electron microscopy, microfluidics, neuronal networks

## Abstract

The human brain contains a wide array of billions of neurons and interconnections, which are often simplified for analysis *in vitro* using compartmentalized microfluidic devices for neuronal cell culturing, to better understand neuronal development and disease. However, such devices are traditionally incompatible for high-pressure freezing and high-resolution nanoscale imaging and analysis of their sub-cellular processes by methods including electron microscopy. Here we develop a novel compartmentalized neuronal co-culture platform allowing reconstruction of neuronal networks with high variable spatial control, which is uniquely compatible for high-pressure freezing. This cryo-fixation method is well-established to enable high-fidelity preservation of the reconstructed neuronal networks and their sub-cellular processes in a near-native vitreous state without requiring chemical fixatives. To direct the outgrowth of neurites originating from two distinct groups of neurons growing in the two different compartments, polymer microstructures akin to microchannels are fabricated atop of sapphire disks. Two populations of neurons expressing either enhanced green fluorescent protein (EGFP) or mCherry were grown in either compartment, facilitating the analysis of the specific interactions between the two separate groups of cells. Neuronally differentiated PC12 cells, murine hippocampal and striatal neurons were successfully used in this context. The design of this device permits direct observation of entire neuritic processes within microchannels by optical microscopy with high spatial and temporal resolution, prior to processing for high-pressure freezing and electron microscopy. Following freeze substitution, we demonstrate that it is possible to process the neuronal networks for ultrastructural imaging by electron microscopy. Several key features of the embedded neuronal networks, including mitochondria, synaptic vesicles, axonal terminals, microtubules, with well-preserved ultrastructures were observed at high resolution using focused ion beam – scanning electron microscopy (FIB-SEM) and serial sectioning – transmission electron microscopy (TEM). These results demonstrate the compatibility of the platform with optical microscopy, high-pressure freezing and electron microscopy. The platform can be extended to neuronal models of brain disease or development in future studies, enabling the investigation of subcellular processes at the nanoscale within two distinct groups of neurons in a functional neuronal pathway, as well as pharmacological testing and drug screening.

## Introduction

Several human brain disorders, including neurodegeneration diseases such as Alzheimer’s (Busche and Konnerth, 2016; Canter et al., 2016; Zott et al., 2018), Huntington’s (Miller and Bezprozvanny, 2010; Miller et al., 2011; Ghiglieri et al., 2019; Blumenstock and Dudanova, 2020) and Parkinson’s diseases (Caligiore et al., 2016; Kim J. et al., 2017; McGregor and Nelson, 2019), have to some extent been attributed to dysfunctions in neural circuitries. Progressive neuronal cell death is thought to be linked in part to abnormal cell-cell communication between subpopulations of neurons in the brain, seen as alterations in synaptic function as well as disrupted intracellular signaling. Particular neuronal subpopulations and specific circuits exhibit selective vulnerability, such as the corticostriatal circuit in Huntington’s disease and the nigrostriatal circuit in Parkinson’s disease, and the entorhinal cortex and hippocampal CA1 projection neurons in Alzheimer’s disease (Saxena and Caroni, 2011). Non-cell-autonomous and circuit-based mechanisms are important to consider in pathogenesis. For example, the excitation of striatal medium-sized spiny neurons is controlled by a combination of glutamatergic inputs from the neocortex and dopaminergic inputs from the substantia nigra pars compacta (Bamford et al., 2004; Cepeda et al., 2007). The cortical projections exhibit early hyperexcitation in Huntington’s disease, and can lead to a toxic convergence of signals onto the striatal medium-sized spiny neurons, thereby enhancing their vulnerability to the effects of the mutant Huntingtin protein (Surmeier et al., 2007). However, the extreme complexity of the brain prevents us from visualizing and monitoring individual neurites or the interactions and communication between specific neurons *in vivo.* Creating cell culture systems that can recapitulate such circuits would be one step closer to mimicking a more physiologically relevant state, and thereby help serve as a platform toward better understanding cellular processes and molecular mechanisms underlying such diseases, and ultimately developing better therapeutic approaches.

An often pursued method toward modeling of neuronal circuitry in culture is compartmentalization. Compartmentalized culturing systems, which are commonly created using microfabrication technology and soft lithography, enable the physical isolation of different cell populations as well as the sectioning of neuronal soma from neurites (Taylor et al., 2005). Various advanced versions of such devices have been used for neurobiological studies of neuron cell development and degeneration, thus capitalizing on the ease of fabrication, reproducibility and cost effectiveness (Neto et al., 2016).

A polydimethylsiloxane (PDMS) device with additional microfluidic local perfusion chambers was fabricated to access synaptic regions with high spatial and temporal resolution (Taylor et al., 2010; Moutaux et al., 2018a). The microfluidic local perfusion chamber provided a novel approach for local manipulation and study of synapse connections between a “presynaptic” and a “postsynaptic” compartment by diffusion of soluble substances. This unique design was also utilized to reconstruct corticostriatal neuronal circuits *in vitro*, providing a microfluidic platform to investigate molecular mechanisms that occur in neuronal circuits, to elucidate pathogenic mechanisms, and to identify potentially effective drug treatments (Moutaux et al., 2018b; Virlogeux et al., 2018). Another example of a compartmentalized microfluidic system for neurobiological cell culture utilizes asymmetric microfluidic channels for unidirectional axonal guidance from a presynaptic sub-population to a postsynaptic sub-population of the neurons to study predefined neuron connectivity *in vitro* (Malishev et al., 2015; Renault et al., 2016; Gladkov et al., 2017; Lassus et al., 2018).

Such compartmentalized microfluidic devices are well-controlled systems compatible with optical microscopy to enable investigation of cellular dynamics with subcellular resolution. However, these devices are often not compatible with vitrification, or cryo-fixation (i.e., avoiding chemical fixatives), by means of high-pressure freezing (Studer et al., 2008) for optimal high-fidelity ultrastructural imaging using approaches such as electron microscopy. Therefore, cellular processes occurring at the nanoscale within such microfluidic devices cannot easily be captured in a near-native state. Currently, cells grown in compartmentalized devices are prepared by more traditional routes for electron microscopy; instead of cryo-fixation, they undergo chemical fixation, subsequent staining using a cocktail of heavy metals to generate high contrast, and dehydration procedures, prior to electron microscopic imaging. Such steps are well-known to potentially introduce artifacts and structural or chemical changes inside biological specimens, which is why cryo-fixation for freeze-substitution and electron microscopy, or cryo-fixation followed by cryo-electron microscopy and tomography, are beginning to take precedence as more high-fidelity approaches for addressing biological questions (McDonald and Auer, 2006).

Cryo-fixation by techniques such as plunge-freezing or high-pressure freezing has emerged in recent decades as the best way to preserve biological samples for ultrastructural studies, i.e., by electron microscopy (Plattner and Bachmann, 1982; McDonald and Auer, 2006; Tsang et al., 2018). Cryo-fixation by high-pressure freezing enables the frozen-hydrated preservation of molecules inside the sample within milliseconds by rapidly chilling of thick samples, generally up to 200 μm in thickness (Studer et al., 2008), to liquid nitrogen temperatures at extremely high pressure (2100 bar). Under these circumstances, the ice nucleation and distortion of specimens during solidification by freezing are minimized: intrinsically contained water turns into vitreous ice even in the absence of cryo-protectants. This results in preserving the cellular architecture (Studer et al., 2001) without requiring the use of any chemical fixatives, hence capturing a near-native, physiologically relevant state. Since this type of freezing occurs on the order of milliseconds, highly dynamic cellular events can be captured with high molecular fidelity. Cells including neurons are established in the literature to vitrify well by high-pressure freezing in systems of similar type (i.e., sapphire disks) (Reipert et al., 2004) without requiring cryo-protectants (Imig et al., 2020). Furthermore, for neuronal cell culture samples that are far thinner, i.e., <10 μm, this falls within a safe range of vitrification by high-pressure freezing, i.e., up to 200 μm in thickness (Studer et al., 2008).

Plunge-freezing also is used to achieve vitreous ice, albeit for thinner samples (<500 nm; typically restricted to macromolecular complexes in solution, or thin cell culture monolayers) as compared to those for high-pressure freezing (up to 200 μm thick samples, such as a wide variety of cell culture or tissues) (Blancard and Salin, 2017). Primary neurons as well as other cell lines have been grown directly atop electron microscopy (EM) grids, which can be plunge-frozen and examined with cryo-electron tomography to study neuronal synapses in a nearly native state (Fernández-Busnadiego et al., 2011; Tao et al., 2018; Liu et al., 2019; Sun et al., 2019). While these systems are useful, typically they utilize only one type of neuron, and further lack any guidance or growth control in a compartmentalized fashion. Indeed, it is very challenging to fabricate a guiding chemical pattern or structure atop EM grids due to size and sensitivity constraints: EM grids are very delicate and relatively small (Ø 3 mm), often containing thin and holey substrates (carbon, formvar) for cell culture compatibility that further complicate them for conventional micro-patterning methods. Micro-contact printing is one approach that has been applied successfully to fabricate protein patterns on EM grids to study the cellular mechanism of dynamic actin cytoskeleton self-organization by electron tomography (Tee et al., 2015). However, after physical contact between the PDMS stamp and the substrate to transfer the protein pattern, the removal of grids from the stamp was found to cause damage to the EM grid surface integrity. Recently, a mask-less photo-patterning has been developed, which can overcome this problem by eliminating physical contact between the stamp and delicate substrate (Strale et al., 2016). This method enables contactless fabrication of protein patterns on EM grids using light-induced molecular adsorption (Engel et al., 2019, 2020; Toro-Nahuelpan et al., 2020). Although this is an innovative approach to create protein micro-patterns on EM grids for biological research, it still requires further development to make it sufficiently suitable for neurobiological research, i.e., of compartmentalized neural circuits.

Among different sample carrier systems for high-pressure freezing, sapphire disks are well suited for cell cultures. They possess sufficient strength to withstand the high pressure during fixation, have high thermal conductivity at low temperature, have sufficient optical clarity for enabling correlative light and electron microscopy studies, and can easily be coated for culturing different types of cells including neurons. Dissociated primary neurons have been shown previously to be cultured on sapphire disks for subsequent downstream processing by high-pressure freezing, freeze substitution, sectioning, and investigation by electron microscopy to investigate subcellular compartments or organelles (Walther et al., 2018). The possibility to capture neurons in their native state also provided snapshots of membrane dynamics at synapse junctions under light stimulation with milliseconds temporal resolution (Watanabe, 2016). However, cryo-fixation methods are not compatible with conventional cell culture set-ups using microfluidic chambers, and there are only limited systems reported which are compatible with neuronal cell cultures.

In our work reported here, we developed a compartmentalized platform, which is uniquely compatible with cryo-fixation by high-pressure freezing followed by freeze substitution to reconstruct compartmentalized neuronal circuits *in vitro* for high fidelity ultrastructural imaging of subcellular structures by methods including electron microscopy. The system allows co-culturing of different populations of neural cells to mimic physiologically relevant neuronal pathways as they exist in the brain, such as corticostriatal or nigrostriatal, or to establish artificial neuronal networks. We fabricated a PDMS device with an embedded microstructured sapphire disk, which is designed in such a way to enable co-culturing of two groups of primary neurons or other neuronal cell lines. The neuritic extensions from the different groups of neurons placed in the two separate compartments are guided by a channel array, which was lithographically fabricated in an SU-8 photoresist on the sapphire disks, and further coated with a suitable neuronal attachment solution. The versatility of our device makes it useful not only for the investigation of ultrastructural features of neuronal networks but also for circuit-level studies of brain diseases and neurological disorders characterized by network dysfunction.

## Results

### Neuron Co-culture System: Device Concept and Fabrication

**FIGURE 1:**
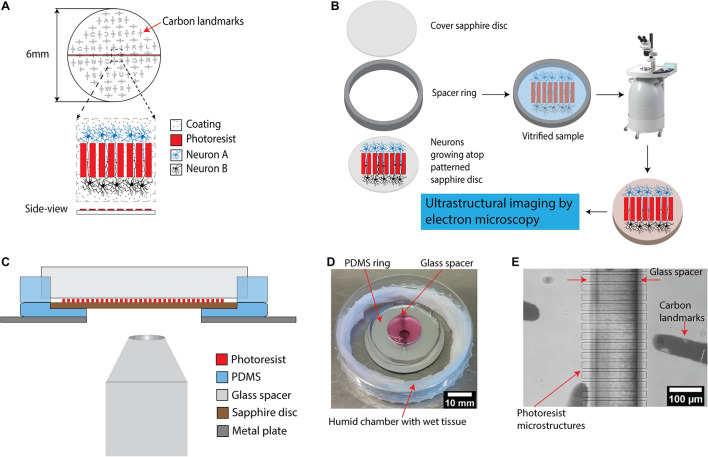
A microstructured device for *in vitro* reconstruction of neuronal networks and ultrastructural imaging. **(A)** Schematic of the microstructured sapphire disks used as cell-growth substrates. Parallel ridge structures are lithographically fabricated on sapphire disks using a biocompatible SU-8 negative tone resist. The structures are guiding neurites originating from neurons of two different populations for neuronal circuits’ reconstruction. An evaporated carbon pattern serves as landmarks in the downstream processing. **(B)** Experimental workflow for ultrastructural imaging: cryo-fixation of cells grown on the structured disks via high pressure freezing, followed by freeze substitution is utilized to preserve cellular ultrastructure for downstream electron microscopy. **(C)** Schematic illustration of an assembled device allowing compartmentalized co-culture of different cell populations. It includes two main components: a PDMS ring with a glass spacer to form two isolated chambers for co-culture, and a PDMS base with a central hole holding patterned sapphire. The whole system is placed on top of a stainless steel ring as a support. The assembled device is compatible with light microscopes to enable correlative light-electron microscopy. **(D)** Picture of an assembled PDMS device. The device is placed into a home-made humidity chamber with a wet tissue to maintain humidity. **(E)** Image of the patterned sapphire disk inside the PDMS device recorded using an inverted microscope. The photoresist ridge structure, the glass spacer separating the two chambers as well as the carbon landmarks were clearly visible.

The central piece of the device presented here consists of a 6 mm sapphire disk with a structure with parallel ridges, used to separate two co-cultured cell populations (Figure 1A). Sapphire disks were selected as the material of choice for a platform base due to their extremely high heat conductance which render them optimal for high-pressure freezing process and their optical transparency required for the light microscopic characterization prior to freezing, i.e., correlative light and electron microscopy (Heiligenstein et al., 2014; Santarella-Mellwig et al., 2018). A finder grid consisting of a thin carbon layer was deposited on the sapphire disk to generate a spatial “map” on the surface for localization of regions of interest for downstream processes including correlation of structures by light microscopy to electron microscopy (Figures 1A,E). The parallel ridge structures with multiple designs were fabricated by standard photolithography on 6 mm sapphire disks (Supplementary Figures 1A–E). SU-8 was chosen as material to fabricate the structures due to its biocompatibility with neuronal cultures (Kim E. et al., 2017). Furthermore, SU-8 is a commonly used material for successfully producing structures with heights of 10–100 μm, and with high aspect ratios. The microchannels divide the center region of the sapphire disks into smaller areas to enable investigation of individual neurites. The length of the microchannels was designed to increase the probability of capturing synaptic connections in the channels between ridges further downstream by electron microscopy. However, it is very challenging to trace if individual neurites from synapses to their respective neuronal cell bodies in long channels because the imaging area of electron microscopy is limited as compared to optical microscopy. Hence, the length of channels was set to 200 μm to balance those requirements. The width of channels was minimized to increase the probability of neurites’ encounters to establish synapse connections between neurons. In addition, narrow channels were utilized to prevent the neurons themselves (soma) from migrating into channels from their respective separated compartments.

Photolithography onto 6 mm sapphire disks is a major challenge, as all the processes from spin-coating to photolithographic exposure are optimized for far larger substrates. Inhomogeneity of the photoresist layer, due to the incompatibility of such small substrates with the standard spin-coating process, prevents its close contact with the photomask during exposure to UV light for crosslinking to ultimately achieve highly resolved microstructures. Therefore, to improve the spin-coating process onto the 6 mm sapphire disks, the disks were mounted into a cavity in the center of a circular sample holder (Ø 15 mm) made from PDMS (Supplementary Figure 2). These perfectly fitting cavities were prepared by placing similar sapphire disks in the center of the molds used for PDMS casting. After mounting, a completely flat surface with an optimally larger surface area (as compared to the small sapphire disk itself) was hence created, which significantly facilitated the spin-coating process to achieve a thin and homogenous layer.

After optimization, ridge structures with minimal distances of ∼10 μm and structure heights of 5–6 μm could be achieved, which is greater than the 3 μm reported as necessary to prevent neurites from crossing over them (Weigel et al., 2012; Supplementary Figure 1F). Patterned sapphire disks were coated with either poly-L-lysine or collagen, which are typical coatings to facilitate the attachment and growth of neuronal cells (Watanabe, 2016; Seven et al., 2020).

For integration of the patterned sapphire disk into a larger device, we fabricated a circular PDMS substrate with a cavity in the center to mount using the same approach as discussed above (Figure 1C). The patterned sapphire disks fit perfectly into the cavity, creating an even, flat surface with an optimally larger surface area. The PDMS material underneath the sapphire disks was removed by puncture to enable direct observation of neurons atop the sapphire disks by inverted microscopy without appreciable light scattering. Moreover, the PDMS circular substrate was fabricated with minimal thickness to reduce the distance between the objective lens of the microscope and the sapphire disk for high-resolution imaging. To stabilize this highly flexible PDMS substrate, it was placed and aligned manually on top of a stainless steel ring, resulting in a minimal working distance of approximately 3.5 mm. Afterward, a PDMS ring divided into two parts by a glass spacer was aligned under a stereo-microscope to cover the microstructures on sapphire disk in order to prevent attachment of the neurons on the patterned area (Figures 1C,E). Usually, the bonding between PDMS parts and substrates is critical for making microfluidic devices. High bonding strength is required to ensure leak-tight encapsulation; for instance, irreversible bonding is achieved by activating the surfaces of both the PDMS parts and the glass substrates with an oxygen plasma and bringing them into contact. However, this type of bonding introduces limitations to internal accessibilities of the devices and inhibits later disassembly, which is required in the present. Alternatively, PDMS substrates can be sealed reversibly to other flat substrates by simply relying on hydrophobic interactions (Taylor et al., 2005; Park et al., 2006; Vitzthum et al., 2010). This concept was followed for our devices, as the glass spacer is the only part with direct contact with the patterned sapphire disk; furthermore, glass does not form direct bonds with photoresist structures.

No culture media leak from the assembled chambers was noted for up to 21 days in culture at 37°C, 5% CO_2_ (Figure 1D). Key features of the assembly such as the glass spacer aligned precisely on top of the photoresist ridge structures and carbon landmarks were visible in an inverted microscope at low magnification (Figure 1E).

The design of this device permits for simple disassembly, and the sapphire disks could be removed from the PDMS chambers easily without damaging the reconstructed neurons and their neurites, for further downstream processes by cryo-fixation via high pressure freezing, followed by freeze substitution and embedding in resin (Figure 1B). The resin-embedded cells were then in an optimal state for being subjected to multiple methods for ultrastructural imaging, i.e., focused ion beam-milling scanning electron microscopy (FIB-SEM) or physical sectioning by a diamond knife for high resolution imaging by transmission electron microscopy (TEM). The same type of physical sectioning by diamond knife could also be easily utilized for serial block-face scanning electron microscopy (SBF-SEM), but this approach was not investigated in this specific study.

### Cell Cultures

#### Culturing PC12 Cell

**FIGURE 2:**
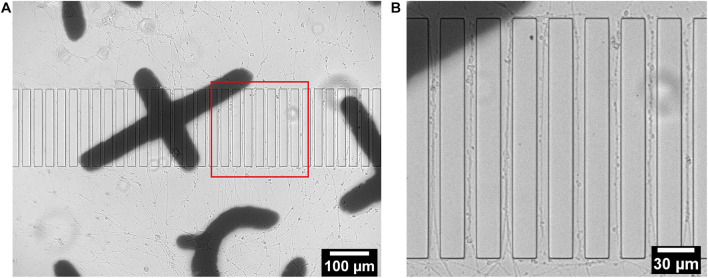
Live imaging of differentiated PC12 cells growing on a structured sapphire disk coated with collagen type IV. **(A)** Projections from differentiated PC12 cells were guided to grow into channels between ridges. **(B)** Zoomed-in red box in **(A)**.

Rat pheochromocytoma cells PC12 are extensively used as models in neuroscience research as they have the ability to exhibit typical neuronal features. In the presence of nerve growth factor (NGF), they undergo neuronal differentiation to adopt the morphology and functionality of neurons with long extensions (Zhou et al., 2006; Martorana et al., 2018; Wiatrak et al., 2020). Before plating into the PDMS device, PC12 cells were labeled with EGFP or mCherry using lentivirus infection in order to enable visualization of potential interactions between two isolated sets of cells. Labeling also facilitated the observation of the response of the neuritic projections of PC12 cells into the micropatterns on the sapphire disks. Labeled PC12 cells were plated into the separated chambers of the PDMS devices, which had been coated with collagen type IV to promote cell attachment and differentiation. During 14 days of differentiation with NGF supplement, the cells were periodically characterized by optical microscopy to monitor the outgrowth of individual neuritic processes. The design of the device permits direct observation of living cells on the sapphire disks inside chambers without the need of disassembling the device. Clear observation of thin neuritic processes by inverted microscopy demonstrates the compatibility of the PDMS device with live microscopic imaging (Supplementary Figure 3). For more detailed analysis of the outgrowth of projections, the patterned sapphire disks were gently removed from the PDMS using tweezers while keeping them immersed in culture media to prevent drying and detachment of adherent cells. The disks were then placed with the structure facing upward in a glass bottom dish containing culture media to enable live cell-imaging using an inverted microscope. The optical images in Figure 2 showed that the PC12 cells were differentiated efficiently and displayed long neurites. Figure 2B clearly demonstrates that the microstructures performed well in directing the neural outgrowth, as multiple neurites were observed to follow patterns and grew in directions outward from each of the two compartments along the ridges.

#### Culturing of Postnatal Hippocampal Neurons

**FIGURE 3:**
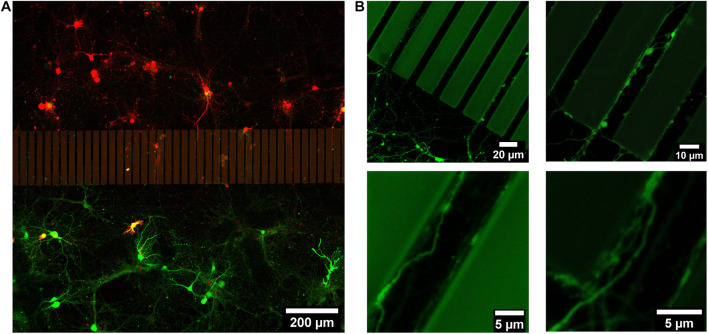
Live imaging of 21 DIV postnatal hippocampal neurons growing on a structured and Poly-L-Lysine coated sapphire disk. **(A)** Two sets of postnatal hippocampal neurons labeled with mCherry and EGFP, respectively, which were growing in separated chambers. **(B)** Representative images of the neurites, which were growing in channels.

Hippocampal neurons were dissociated from postnatal mice hippocampi. Two groups of dissociated neurons were incubated with lentivirus to fluorescently label them with either EGFP or mCherry prior to cell seeding. Those labeled groups of neurons were introduced into separated areas on the patterned sapphire disks in the PDMS devices. Hippocampal neurons exhibited good viability at 21 DIV, confirming the biocompatibility of the SU-8 photoresist with such primary neuronal cultures. The viability of neurons and the outgrowth of neurites on the patterned sapphire disks were investigated using inverted optical microscopy (Supplementary Figure 4). The glass spacer covering microstructures was also visible by optical microscopy. As shown in Figure 3, the intact neuronal networks with fine extending neurites are imaged at higher resolution by confocal fluorescence microscopy after disassembling them to investigate neurites in the microchannels. Labeled neurons that were expressing EGFP or mCherry grew inside the separated compartments on the patterned sapphire disk, and were efficiently separated by the ridge structure and the glass spacer (Figure 3A). The compartment-specific expression of either EGFP or mCherry demonstrated the ability of our device to introduce and maintain independent culturing of two sets of different cells in compartments separated by the patterned microstructures on the patterned sapphire disks. This permits the investigation of the interactions between the two cell populations. It was observed that neuritic processes extending from neuronal cell bodies followed along the micropatterned channels (Figure 3B). Red or green fluorescent neurites extending from either mCherry- or EGFP-labeled neurons, respectively, were evident within the microchannels, and neurites that contacted the inner walls of the micropatterned channels continued to grow parallel along the length of that wall toward the neighboring compartment, rather than climbing upward and over the ridges. It is likely that the limited flexibility of microtubules and actin filaments hindered the significant bending of growth cones’ filopodia to change to a more perpendicular direction of outgrowth (Dunn and Heath, 1976; Mahoney et al., 2005).

#### Culturing of Embryonic Striatal Neurons

**FIGURE 4:**
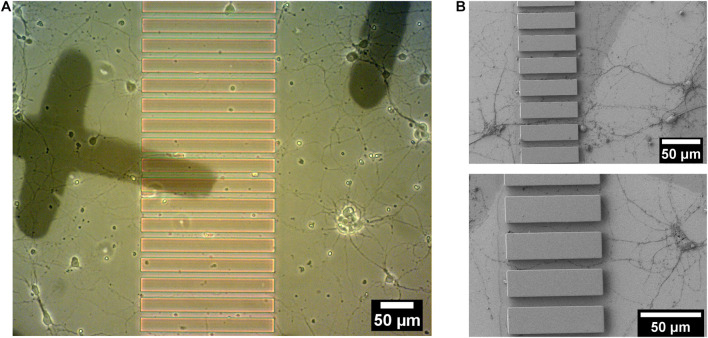
14 DIV embryonic striatal neurons which were growing on Poly-L-Lysine coated-patterned sapphire disk. **(A)** Live imaging showed neurites from neurons on both sides of the structure growing into channels. **(B)** SEM images of the neurites, which were growing into channels. Crossing of neurites over the ridge structures was not observed in any sample.

Striatal neurons from embryonic (E18) rat striatum were cultured on Poly-L-Lysine coated patterned sapphire disks with an optimized density (160 cells/mm^2^). Growing striatal neurons were observed by live imaging using an optical microscope at 14-DIV to localize regions of interest shortly before the cryo-fixation process by high-pressure freezing, to prepare such samples for downstream electron microscopy. Neurons demonstrated good viability and the neurites from different neurons were observed to successfully enter the microchannels between ridges from both directions (Figure 4A). The individual channels, in which neurites from opposite sides of microchannels established the contacts, were targeted for downstream analysis. The landmarks produced from evaporated carbon were stable and clearly visible after 14 days in culture. They were used to precisely identify regions of interest by optical microscopy for subsequent processing. Furthermore, we found that microstructures provided sufficient geometrical guidance cues to direct neuritic outgrowth from the striatal neurons into the predefined patterns. None of the observed neurites crossed over the ridges of the microchannels. This was confirmed in another set of experiments performed by scanning electron microscopy (SEM), which enabled imaging the neuronal networks at higher resolution compared to optical microscopy. In this case, the striatal neurons growing on the structured sapphire disks were chemically fixed, followed by dehydration by a series of ethanol concentrations in distilled water, drying in a critical point drier and Au coating for SEM imaging. SEM images in Figure 4B clearly showed that the neuritic extensions from the neurons grew only within the channels. No visible neurites appeared crossing over, or on top, of the ridges of the microchannels. This finding is consistent with previous reports that the ridges of structures with heights over 3 μm was sufficient to prevent neurite overcrossing (Weigel et al., 2012).

#### Ultrastructural Imaging

##### Focused ion beam – scanning electron microscopy (FIB-SEM)

After cryo-fixation via high pressure freezing, 14 DIV E18 striatal neurons were subjected to a freeze substitution process using a substitution solution containing heavy metals for staining (to enhance contrast for electron microscopy) followed by resin embedding (for details see section “Materials and Methods”). The resin blocks were then processed by FIB-SEM to investigate the ultrastructure of the embedded neuronal networks. FIB-SEM is a commonly used method for 3D biological imaging developed in the last decade (Knott et al., 2008; Narayan and Subramaniam, 2015; Xu et al., 2017). Typically, the 3D FIB-SEM procedure utilizes a focused ion beam to sequentially remove thin layers (a few nanometers thick) of either frozen-hydrated or resin-embedded samples to expose a fresh surface for subsequent imaging by SEM. This enables the 3D reconstruction of samples with nanoscale resolution, albeit relatively less than that afforded by transmission electron microscopy (TEM).

**FIGURE 5:**
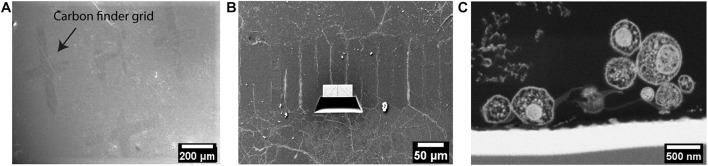
Ultrastructural analysis of resin-embedded embryonic striatal neurons by FIB-SEM. **(A)** SEM images of the surface of resin block. The carbon finder grid was transferred to the resin block and it was clearly detectable by SEM. **(B)** The neurites in the channels were targeted and processed by FIB-SEM. **(C)** Cross-section of neurites in a channel laid open by FIB and imaged by SEM.

As there is very little contrast between photoresist structures and the embedding resin, the parallel-ridge structures of the microchannels are not directly visible in the SEM. However, following freeze substitution and resin embedding, and after removal of the sapphire disk the carbon landmarks were found to be transferred to the resin block and were clearly visible by SEM (Figure 5A). Furthermore, the patterned regions were recognized easily by observing the neuritic outgrowths into the channels between the ridges (Figure 5B). Numerous neurites originating from opposite sides of the microstructures were clearly visible within the separating channels. Using the carbon evaporated landmarks, the regions of interest selected earlier by optical microscopy were targeted and processed by FIB, to reveal the embedded neurites within microchannels. The freshly milled surface was captured by SEM and the internal ultrastructure of multiple neurites within the channels were clearly revealed (Figure 5C). The neurites were optimally embedded within the resin block and located very proximally (within 70 nm ∼ 300 nm) to the surface, which actually represents the original interface to the sapphire disk. The SEM images also showed various neurites entering a single channel and establishing close physical contacts. The excellent resolution provided by FIB-SEM is well suited to study the ultrastructure of such embedded neuronal networks. However, the process time is a limiting factor, as it would take approximately 1–2 weeks to investigate a single 200 μm-long channel at high spatial resolution. Therefore, such a method appears more suitable to obtain high-resolution images for pre-targeted regions, rather than scanning or “fishing” generally for synaptic connections within a long channel, for example.

##### Serial sectioning – transmission electron microscopy (TEM)

The resin blocks that contained embedded embryonic striatal neurons were physically sectioned in an ultramicrotome using a diamond knife to prepare ultrathin sections (50–60 nm thickness) for TEM imaging. The channels between the patterned ridges were targeted for processing to reveal the ultrastructure of neurites within. Compared to FIB, such ultramicrotome sectioning enabled cutting along the length of microstructures and a large area could be captured in a single section. Thus, substantially larger imaging areas were possible to obtain, allowing us to visualize and track neurites within the channels with relatively less processing time. However, it was challenging to capture the neurites in these ultrathin sections because those were accumulated very near to the surface of the resin block.

**FIGURE 6:**
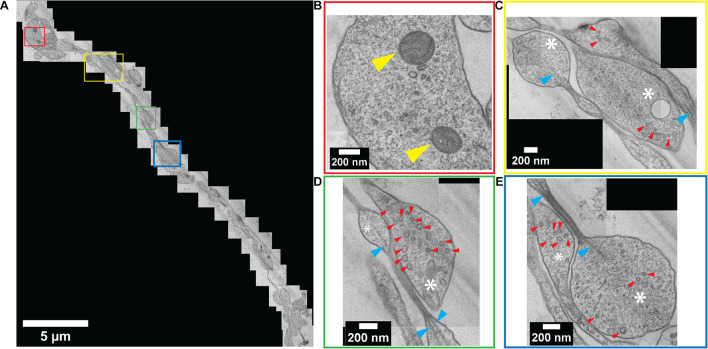
Ultrastructural analysis of resin-embedded embryonic striatal neuron obtained by serial sectioning – TEM. **(A)** A set of TEM micrographs of neurites embedded in a single channel. **(B–E)** Enlarged images of boxes outlined in **(A)** with corresponding colors. Yellow arrowhead, mitochondria; white asterisk, axonal bouton; red arrowhead, synaptic vesicle; blue arrowhead, microtubule.

Standard slot-type electron microscopy grids were used to collect the ultrathin sections to ultimately enable a larger viewing window by TEM imaging. Numerous neurites were observed within a single channel (Figure 6A). TEM images revealed a perfectly preserved ultrastructure of the embedded neurites with high contrast. Colored boxes in Figure 6A represent areas that are zoomed-in to show a higher magnification in Figures 6B–E. At this magnification, internal cellular structures including microtubules, synaptic vesicles, and mitochondria are clearly visible. Axonal terminals are also recognizable based on their button-like shape (white asterisk in Figures 6C–E). Multiple physical contacts between neurites were observed to be established in the channel area. However, no ultrastructural evidence of a synapse was observed in this particular channel.

## Discussion

Here we describe a compartmentalized platform enabling ultrastructural imaging of neuronal co-cultures, akin to those separated by channels in microfluidic devices to recreate neuronal pathways and circuits. While the application of high-pressure freezing (Studer et al., 2014) and freeze-substitution of neurons (Sosinsky et al., 2008; Imig et al., 2020) are well-established in the literature, the novelty of our approach is in the application of our device system for enabling co-culturing, akin to microfluidics, which is of valuable functional importance in neurobiological research. We demonstrate topographical structuring of sapphire disks as a successful strategy to control cellular adhesion and growth. The fabrication of micropatterned channels atop the sapphire disks by an adapted photolithography process enables the selective guidance of neurites to the adjacent compartment housing compartmentally distinct neurons, all of which can be cryogenically fixed in a near-physiological state in vitreous ice to preserve their structure for ultrastructural studies, while avoiding the use of chemical fixatives and dehydration. We have shown that a variety of neuronal-type cells can be grown and imaged successfully using our platform: neuronally differentiated PC12 cells and both murine hippocampal neurons and striatal neurons. Our results set a solid foundation for next steps in terms of culturing two distinct neuronal populations for re-creating more complex neuronal pathways/circuits, such as the corticostriatal and nigrostriatal pathway. For example, one compartment can be used for culturing cortical cells, and the other compartment can be used for culturing striatal cells. We have successfully demonstrated using our EGFP/mCherry studies that the two distinct neuronal populations do not cross over the channel barrier, and only make contact via their extending neurites through the micropatterned channels. This should open the door to more physiologically relevant discoveries at the nanoscale level using such cryo-fixed, vitrified neuronal networks for high-fidelity electron microscopy.

Since photolithography is a well-established patterning method that enables large-scale mass production and high reproducibility, the current fabrication process could be feasibly scaled up by means of designing a sample holder to allow parallel processing of multiple samples at the same time. In future developments it would be useful to enhance the resolution of the microstructures for reconstruction of neuronal networks. For instance, smaller microchannels (2–3 μm) are more effective in completely preventing the migration of the neurons themselves into the channels, as well as limiting the number of neurites entering the channels. This enables more clear and distinct tracing of individual neurites to their respective neuron as well as the synaptic contacts downstream. More elaborate microstructures with higher resolution could be utilized for creating unidirectional patterns for the investigation of predefined neuronal connectivity (Malishev et al., 2015; Renault et al., 2016; Gladkov et al., 2017; Lassus et al., 2018) or the addition of extra chambers for local manipulation of neuronal circuits (Taylor et al., 2010; Moutaux et al., 2018a). Since the resolution of fabricated microstructures on sapphire disks is mainly limited by the incompatibility of small samples with the standard photolithography processes, other new procedures can be used to overcome this barrier. Specifically, resolution can be increased using recent advanced mask-less lithography techniques, which allow exposing the patterns directly onto the resist-covered surfaces and can enable the fabrication of 2D or 3D microstructures with high resolution on small substrates (Guijt and Breadmore, 2008; Braun and Maier, 2016; Varapnickas et al., 2020).

An important feature of the developed devices is their compatibility with optical microscopy as well as downstream electron microscopy, thereby enabling the correlative study of neuronal networks. By optical microscopy, it is possible to monitor the live imaging of growing cells on top of patterned sapphire disks and the outgrowth of neuronal processes into the microchannels while the sapphire disks are still retained within the PDMS chambers. The neurites present in the microchannels can be live-imaged distinguishably via the expression of distinct fluorescent proteins, for example (Figure 3B). As a result, distinctly labeled neurites originating from different groups of cells can be traced and analyzed to study specific cell-cell communications as well as their responses to topological cues. The information obtained from optical microscopy can be precisely correlated to the electron micrographs of the same structures for interpretation of dynamic events and their ultrastructure at the nanoscale.

We have cultured multiple neuronal cell lines, including primary striatal and hippocampal neurons successfully on our platform for up to 3 weeks. All cell lines exhibit similar viability as in control experiments on culture dishes, confirming the biocompatibility of our platform for neuronal cell cultures. We also demonstrated the ability of microstructures in guiding the distal projections from separately growing groups of neuronal cells to form physical contacts (Figures 2–4). Although we have not proven the formation of synapses between the neurons, we have shown that neurites make physical contact as shown in Figures 2, 4–6, supporting the idea that such microstructures can be applied to image synapses. Given the versatility of our device, future work will undoubtedly include neurons from different brain regions cultured in the distinct compartments of our device, in order to study physiologically relevant neuronal circuits to neurodevelopment and neurodegeneration, for example. Moreover, since each neuronal population has been demonstrated to be distinctly maintained in each component, future experiments could enable manipulations to individual cells in either component as part of a functional compartmentalized network, i.e., pharmacological, chemical or genetic interventions by methods such as single cell transfection or microinjection (Chow et al., 2016) or nanofluidics atomic force microscopy (Meister et al., 2009), which allows the injection of chemical molecules or drugs into single cell(s). For example, the neurotoxin 1-methyl-4-phenylpyridinium (MPP+), the active metabolite of 1-methyl-4-phenyl-1,2,3,6-tetrahydropyridine, can be applied to one of the two compartments of neurons to induce neuronal death as relevant to Parkinson’s disease, for correlative biochemical and ultrastructural studies regarding the degeneration of nigrostriatal dopaminergic neurons (Zeng et al., 2006; Ito et al., 2017; Risiglione et al., 2020). Furthermore, the responses of neuronal cells to pharmacological agents, such as neuroprotective agents, in the context of disease models can also be investigated at the ultrastructural level in a near-native state to validate their potentials in brain diseases’ treatment (Zeng et al., 2006; Peng et al., 2013; Guo et al., 2019).

Following cryo-fixation by high pressure freezing and subsequent freeze substitution, the resulting resin-embedded neuronal networks are compatible with multiple methods to study their ultrastructure by electron microscopy. FIB-SEM and serial sectioning-TEM were applied successfully to image the well-preserved ultrastructure of neuronal networks at high resolution (Figures 5, 6). For future studies, the ultrathin sections prepared by serial ultramicrotome sectioning can be combined with immunolabeling to allow the localization of macromolecules at high resolution in the cellular context (Lucocq, 1994; Schwarz and Humbel, 2007; Flechsler et al., 2020). Theoretically, another approach known as serial block-face scanning electron microscopy (SBF-SEM) would enable the imaging and investigation of neurites inside the microchannels with a relatively larger field of view as compared to FIB-SEM. Thus, it would allow for a more efficient, wide-field view of imaging and investigation of neurites that grow inside the microchannels. However, the large ratio of non-conductive embedding resin of our samples caused charging problems during our attempt at SBF-SEM imaging. Future work that utilizes a more conductive resin during the freeze-substitution process would theoretically eliminate such charging artifacts that we had encountered, thereby improving the imaging by SBF-SEM (Nguyen et al., 2016; Thai et al., 2016; Yoshimura et al., 2017, p. 1; Takeda et al., 2018). Future endeavors are also envisioned to capitalize on our device’s ability for enabling live cell CLEM (van Rijnsoever et al., 2008; Fermie et al., 2018), or probing fluorescent labeling in-resin (Luby-Phelps et al., 2003; Lucas et al., 2014; Tanida et al., 2020), within a more physiologically relevant system: one that is microfluidic-based and thereby recapitulates a distinct two-way neuronal network, accessible for the first time by cryo-fixation. Beyond this, our system could be adapted for research questions of a wider scope, such as cell migration and cell sorting, and extended to different types of sample carriers for high-pressure freezing, which would directly be compatible for cryo-ultramicrotomy and cryo-electron microscopy and tomography, either by cryo-FIB-SEM or cryo-TEM.

## Conclusion

Our cell culture platform with distinct compartments connected by microchannels akin to microfluidics, is uniquely compatible for high-pressure freezing for chemical-free preservation in a near-native vitreous state. It can thereby enable the recapitulation and imaging of neuronal pathways and circuits, and opens the door to more physiologically relevant nanoscale imaging, and cryo-electron microscopy and tomography of compartmentalized neuronal “microfluidic”-type cultures, which was previously unachievable due to technical constraints that we have overcome. Our device can be expanded to studying neuronal systems in the context of disease and drug testing for correlative studies and high-resolution imaging, thereby serving as a tool of great potential to neuroscience research.

## Materials and Methods

### Fabrication of Microstructures on Sapphire Disks

Polymeric microstructures for neurite outgrowth guidance were lithographically fabricated on 6 mm sapphire disks (Leica Microsystems, Switzerland), which were cleaned with Piranha solution (2:1 vol H_2_SO_4_ : H_2_O_2_) at 90°C for 1 hr. The disks were then landmarked by carbon evaporation (MED010, BalTec AG, Switzerland) through a finder grid mask (Leica Microsystems, Switzerland) and baked at 190°C for 6 h to stabilize the carbon pattern. A 6 μm layer of SU-8 photoresist (SU-8 5, MicroChem Corporation, Newton, MA, United States) was spin-coated on the sapphire disks at 500 rpm (200 rpm/s acceleration) for 5 s and 2,000 rpm for 40 s (500 rpm/s acceleration). It was soft baked at 65°C for 1 min, 95°C for 3 min and then exposed at 365 nm wavelength at a dose of 100 mJ/cm^2^ (Karl Suss, MJB3) using a chromium mask produced with standard e-beam lithography. Following a post expose bake at 65°C for 1 min and 95°C for 1 min the structures were developed in AZ^®^ EBR Solvent (Microchemicals GmbH, Germany) for 1 min and hard baked at 190°C for 30 min on a hot plate to further crosslink the SU-8 photoresist. The final profile of the structures was measured using a Veeco Dektak 8 profilometer.

### Fabrication of the PDMS Chamber

The device to enable co-culture of neurons in separate areas on sapphire disks (Figure 1) consists of components which were fabricated in PDMS (Sylgard 184, Dow Corning) using a mixture of silicone elastomer and its curing agent in a ratio of 10:1. Crosslinking was done at 80°C for 1 h. A circular PDMS substrate (Ø 30 mm, thickness 3 mm) was fabricated, using a sapphire disk as a mold to create a cavity that fits perfectly to sapphire disk. It was then punctured to create a circular hole (Ø 4 mm) concentrically to the Ø 6 mm cavity to enable neuron live-imaging on an inverted microscope. This substrate was placed manually on top of a stainless steel plate (Ø 35 mm, thickness 0.2 mm) with a circular hole (Ø 4 mm) in the center prepared by laser cutting. The PDMS substrate and metal plate were manually aligned using their Ø 4 mm holes. The patterned sapphire disk was placed into the Ø 6 mm cavity of circular PDMS substrate. A PDMS ring (inner Ø 15 mm/outer Ø 30 mm, thickness 3 mm) with a square glass spacer (20 mm × 7 mm, thickness 160–190 μm) in the center to divide it into two separated chambers was placed on top of the PDMS substrate containing patterned sapphire disk under a stereo microscope and the glass spacer was aligned to cover the parallel ridges of the photoresist structure. The assembled device was oxygen plasma treated for 1 min and sterilized by 70% ethanol. To promote attachment and growth of neurons 0.15 ml of 0.01% Poly-L-lysine solution (Catalog # 3438-100-01, Trevigen, Gaithersburg, MD, United States) was added to the chambers and incubated for 2 h at room temperature (0.15 ml/cm^2^). The device was then washed three times with H_2_O, dried under the tissue culture hood and stored at 4°C until neuron culture. Alternately, for PC12 differentiation experiments, the device was coated with collagen type IV (C5533, Sigma-Aldrich) reconstituted in sterile 0.25% acetic acid. After sterilization by EtOH, 600 μl of 0.023 mg/ml collagen type IV solution was added to PDMS device and incubated over night at 37°C. The coating solution was then removed and device was airdried, and stored at 4°C until use.

### Cell Cultures

Primary striatal neurons were dissociated from E18 rat embryo brain tissue (BrainBits LLC, United States) and hippocampal neurons were dissociated from P0 mouse pups (courtesy of E. Pecho-Vrieseling, University of Basel, Switzerland). The dissociation was performed according to detailed protocols from BrainBits LLC^1^. The dissociated neurons were seeded onto the samples at an optimized density (16.000 cells/cm^2^ for embryonic hippocampal neurons and 32.000 cells/cm^2^ for postnatal hippocampal neurons) in NbAct1 media (BrainBits LLC, United States) and were grown for up to 21 days in a humidified cell culture incubator with 5% CO_2_ at 37°C.

PC12 cells were obtained from ATCC (Catalog # CRL-1721) and cultured in DMEM media supplemented with 10% heat-inactivated horse serum, 5% fetal bovine serum and 1% penicillin-streptomycin. Cells were cultured in poly-l-lysine coated petri dishes (0.01% solution, 0.15 ml/cm^2^) and the culture medium was refreshed every 3 days. For differentiation experiments, PC12 cells were harvested and transferred to a collagen coated PDMS device in DMEM media supplemented with 1% heat-inactivated horse serum, 1% penicillin-streptomycin and 100 ng/ml NGF at cell density 1,000 cells/cm^2^. PC12 cells cultures were maintained at 37°C, 5% CO_2_ and the media was replaced every 2 days.

The growing cells were periodically checked by inverted microscopy, using 20× and 40× lenses. After cells were growing for a defined period of time, the PDMS device was disassembled and sapphire disk was removed from PDMS substrate. Cells cultivated on the sapphire disks were then fixed by standard chemical means or high pressure freezing for downstream investigation.

### Lentiviral Labeling

HEK293T cells were plated at a density of 50% in DMEM media containing 10% fetal bovine serum and 1% penicillin-streptomycin in 175 cm^2^ cell culture flask 24 h before transfection. A mixture of 24 μg lentiviral transfer plasmid pLV-eGFP (#36083, Addgene, United States) or 24 μg pLV-mCherry (#36084, Addgene, United States) with 8 μg each 3rd generation viral packaging vectors: pMDLg/pRRE (Addgene plasmid # 12251) pRSV-Rev (Addgene plasmid # 12253) pVSV-G (Addgene #138479) was prepared. The DNA mixture and PEI (branched polyethylenimine, Sigma-Aldrich, 1 mg/ml) were mixed at the ratio of 1:3 in a total volume of 2 ml of OptiMEM media (Gibco) and incubated at room temperature for 10 min to form complex before transfection. Then, the mixture was added to the cells to initiate the transfection.

The supernatant containing virus from transfected HEK293T was harvested at 48 and 72 h post transfection in a combined harvest where all the individual harvests were pooled. Each harvested media was stored at 4°C between harvests. The viral supernatant was centrifuged at 200 × *g* for 5 min at room temperature to pellet any cell debris that was collected during harvesting. Then, it was filtered through a 0.45 μm filter and concentrated by Polyethylene Glycol (PEG) precipitation. The PEG solution was prepared by dissolving 85 g PEG 6000, 17.5 g NaCl in 25 ml 10× PBS and 100 ml sterile water and stored at 4°C until use. The PEG solution was added to filtered virus supernatant at ratio 1:3 and final PEG and NaCl concentration will be 8.5% and 0.3M, respectively. After gently mixing, it was stored overnight at 4°C. Next, the mixture was centrifuged at 1,600 × *g* for 1 h at 4°C. After gently aspirating the liquid, the pellet was resuspended in 1/250 of the original volume of cold PBS. The virus suspension was aliquoted and stored at −20°C. For transduction of PC12 cells, concentrated virus suspension was added to undifferentiated PC12 cells, which were growing in poly-l-lysine coated petri dishes. Two days after transduction, PC12 cells were harvested and transferred to a collagen coated PDMS device for differentiation.

For transduction of primary neurons, following dissociation, the concentrated virus was immediately added to neuron suspension in NbAct1 media (BrainBits LLC, United States) and incubated for 1.5 h at 37°C, 5% CO_2_. After that, the cells suspension was centrifuged at 1,100 rpm for 3 min to remove virus and cells pellet was resuspended in warm NbAct1 media (BrainBits LLC, United States). The infected neurons were seeded onto PDMS device, grown for up to 21 days, and imaged by confocal microscopy.

### Optical Microscopy

For imaging of cells in the assembled chambers we used a FLoid^TM^ Cell Imaging Station from Thermo Fisher Scientific. Confocal fluorescence microcopy and high resolution imaging in wide field/phase contrast mode were performed on a Nikon Eclipse Ti2 microscope, which is equipped with a Nikon Confocal A1 HD25 unit, using CFI Plan Apochromat Lambda lenses with 10× to 40× magnification.

### SEM Imaging of Neuron Cultures to Investigate Neural Outgrowth on Microstructured Sapphire Disks

Neurons were chemically fixed by 4% paraformaldehyde diluted in filtered DPBS pH 7.4 for 10 min at room temperature. Cells were then rinsed three times with DPBS, three times with distilled water. Dehydration was performed through a graded series of ethanol beginning with 25% EtOH (1 × 5 min), followed by 50% EtOH (1 × 10 min), 75% EtOH (1 × 10 min), 95% EtOH (1 × 10 min), and 100% EtOH (3 × 10 min). The samples were then quickly transferred in 100% EtOH to critical point dryer (EM CPD300, Leica Microsystems, Switzerland) for drying by liquid CO_2_. Neurons always remained immersed in liquid in between the washing steps and during transfers to avoid any intermediate drying. After critical-point drying, the samples were sputter coated with ultrathin gold layer (SPI-Module Sputter Coater, Structure Probe, Inc., United States) and imaged by Scanning Electron Microscopy (ZEISS NVision 40).

### High Pressure Freezing

Sapphire disks with growing neurons were mounted face-up into a plastic 6 mm middle plate, and a metal spacer ring (Ø 6 mm, 100 μm thickness) was then placed on top. Before capping the assembly with another bare sapphire disk to form a sandwich, a droplet of neuron culture medium was added in order to protect the neurons in a small liquid chamber. The sandwich was then simultaneously pressurized at high pressure (2100 bar) and frozen at liquid nitrogen temperatures using a Leica EM ICE high pressure freezer (Leica Microsystems, Vienna, Austria). Frozen samples were stored in liquid nitrogen for downstream processing and imaging.

### Freeze Substitution

Neuronal cell cultures on sapphire disks were high pressure-frozen at PSI, then transported in liquid nitrogen to ScopeM (ETH Zurich). Samples were subjected to freeze-substitution in acetone, dried over molecular sieve and supplemented with 0.5% uranyl acetate (Polysciences; added from a 5% stock solution in methanol) and 1% osmium tetroxide (Polysciences). 5% double distilled water was added to the FS-cocktail, to improve visibility of membranes (Walther and Ziegler, 2002). The samples were kept at −90°C for 3 h, then warmed gradually to −20°C at 5°C/h, left at −20°C for 2 h, then warmed to 0°C at 10°C/h, and left 1 h at 0°C, before bringing them to room temperature and letting them adapt for 1 h. Then, samples were rinsed twice in dry acetone, microwave-assisted (Pelco BioWave, Ted Pella Inc., Redding, CA, United States). For resin embedding, the samples were infiltrated using 30% Epon (Fluka Epoxy Embedding Kit) in dry acetone twice, microwave-assisted, followed by two changes of 70% Epon. Then samples were subjected to three changes of 100% Epon for 1.5 h each, at RT on a shaker, before transferring them into molds with fresh resin and polymerization for 3 days at 60°C. After polymerization, sapphire disks were detached by briefly dipping the warm resin block into liquid nitrogen, leaving the cells embedded in the resin block.

### Ultrastructural Imaging

For FIB-SEM (NVision 40, Zeiss), the resin block was trimmed to approximately 5 mm height and glued to a SEM-stub using silver conductive epoxy glue (CircuitWorks; Chemtronics, Hoofddorp, Netherlands) and allowed to dry for 1 day at room temperature. Identification of a ROI and FIB-SEM imaging was performed as previously described in Lucas et al. (2017) (see section “Materials and Methods”). Briefly, by increasing the acceleration voltage to 15 kV and thus increasing the interaction volume of the electron beam with the sample, backscattered and secondary electrons could be detected selectively from within the resin block, revealing the cells stained with heavy metal salts for precise positioning of the trench-milling. Milling a trench to expose the cells for imaging and imaging conditions were applied as described (Lucas et al., 2017).

For TEM, small series of ultrathin sections (50–60 nm thickness) were obtained with a diamond knife (Diatome Ltd., Switzerland) on a Leica UC6 ultramicrotome (Leica Microsystems, Vienna, Austria), collected atop Formvar- and carbon-coated slot grids (Quantifoil, Großlöbichau, Germany), and subsequently stained with 2% aqueous uranyl acetate for 5 min, and Reynold’s lead citrate for 30 s. Stained sections were then visualized using a Morgagni 268 TEM at 100 kV (FEI Company, Eindhoven, Netherlands). As the neuronal cells and their neurites can cover an area of several hundreds of microns, overlapping images were collected that follow neuritic processes at high magnification. These image series were stitched into one large image using the TrakEM plugin of Fiji (Schindelin et al., 2012).

## Data Availability Statement

The original contributions presented in the study are included in the article/Supplementary Material, further inquiries can be directed to the corresponding author/s.

## Author Contributions

SS devised the project and obtained project grant funding. SS, CP, and HT planned the experiments and interpreted the results. HT designed and manufactured the device, performed cell culturing, lentivirus preparation and transfection, chemical fixation, SEM characterizations, and high-pressure freezing with advice by SS, and made all figures and schematic models, and wrote the manuscript together with CP and SS, with input from all other authors. HT, TI, and ML built the strategy of freeze substitution and resin embedment of frozen cell cultures, ultramicrotomy, and TEM imaging of ultrathin sections. ML performed the strategy. SS, CP, and TI supervised HT. TI further supported HT in project logistical needs. All authors contributed to the article and approved the submitted version.

## Conflict of Interest

The authors declare that the research was conducted in the absence of any commercial or financial relationships that could be construed as a potential conflict of interest.

## Publisher’s Note

All claims expressed in this article are solely those of the authors and do not necessarily represent those of their affiliated organizations, or those of the publisher, the editors and the reviewers. Any product that may be evaluated in this article, or claim that may be made by its manufacturer, is not guaranteed or endorsed by the publisher.

## Footnotes

1
www.brainbitsllc.com
