# Supplementary material for: A Compartmentalized Neuronal Cell-Culture Platform Compatible With Cryo-Fixation by High-Pressure Freezing for Ultrastructural Imaging
Source: Front Neurosci. 2021 Sep 8;15:726763. doi: 10.3389/fnins.2021.726763 (PMC8455873; doi:10.3389/fnins.2021.726763)
Supplement: Supplementary file 1 [file Data_Sheet_1.PDF]

Supplementary Material

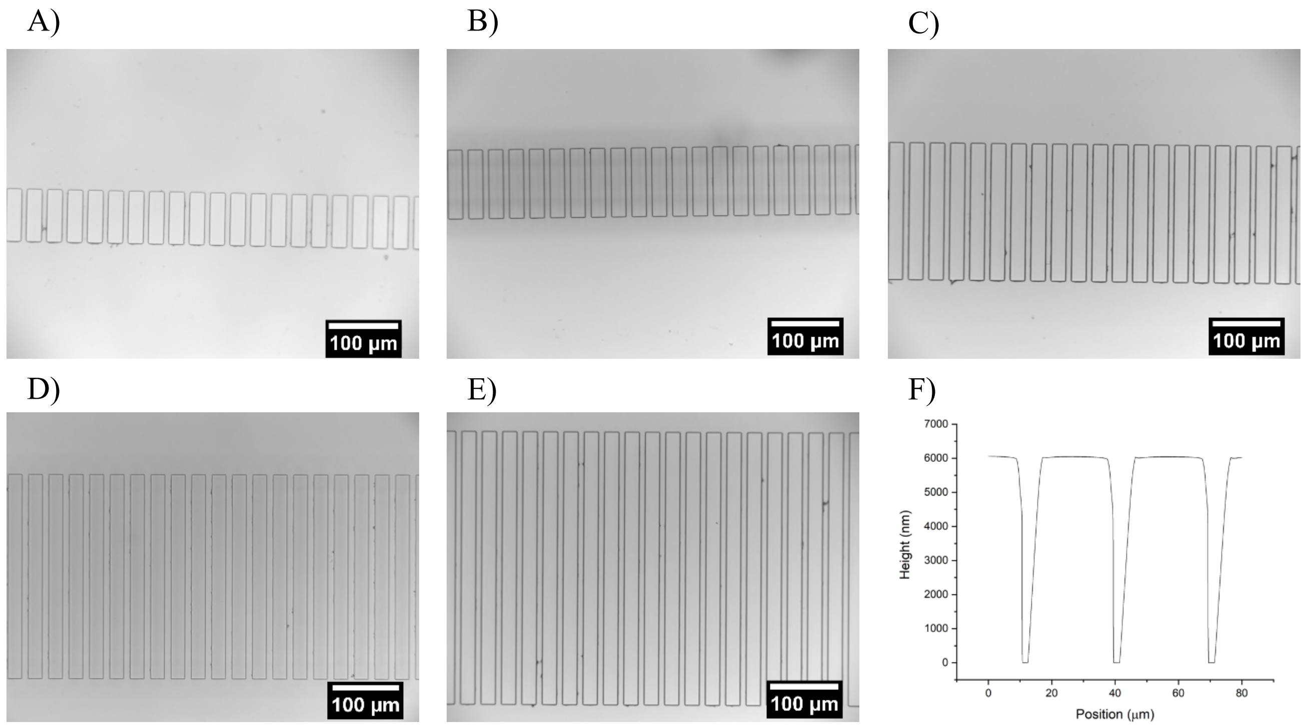

Supplementary Figure 1. (A) Length: 75 µm, Width: 20 µm, Spacing 10 µm; (B) Length: 100 µm, Width: 20 µm, Spacing 10 µm; (C) Length: 200 µm, Width: 20 µm, Spacing 10 µm; (D) Length: 300 µm, Width: 20 µm, Spacing 10 µm; (E) Length: 400 µm, Width: 20 µm, Spacing 10 µm; (F) Thickness of the microstructures, measured by profilometer.

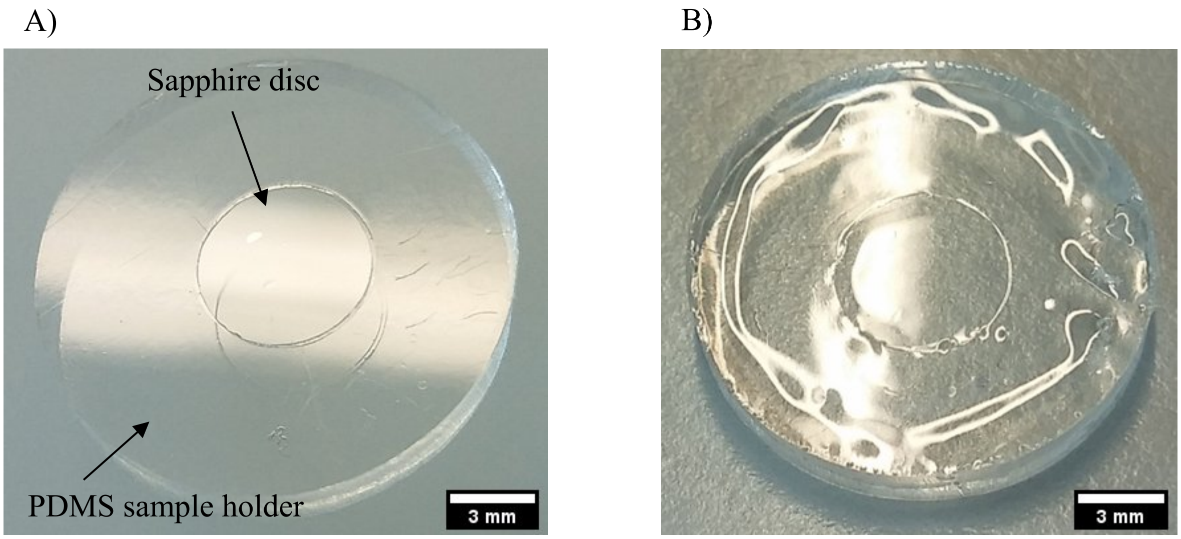

Supplementary Figure 2. A sample holder to improve the spin-coating process on the 6mm sapphire discs. (A) Sapphire disc inside the PDMS sample holder. (B) Spin-coated SU-8 photoresist layer on sapphire disc with support of PDMS sample holder.

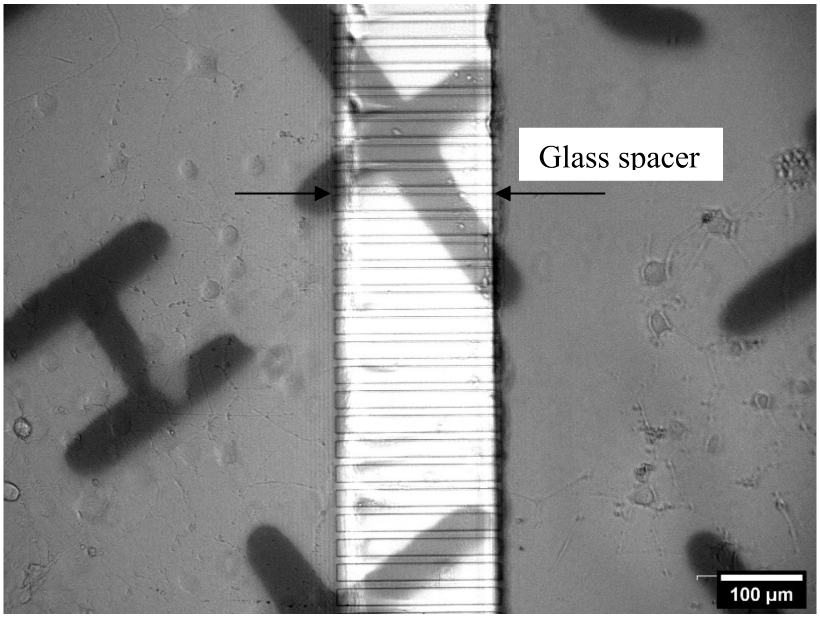

Supplementary Figure 3. Live imaging of 5 DIV differentiated PC12 growing on a structured and collagen coated sapphire disc inside PDMS chamber. 5 DIV differentiated PC12 cells growing on patterned sapphire disc with long extensions. The glass spacer which was aligned to cover microstructures is visible (at the tip of the arrows).

A) B)

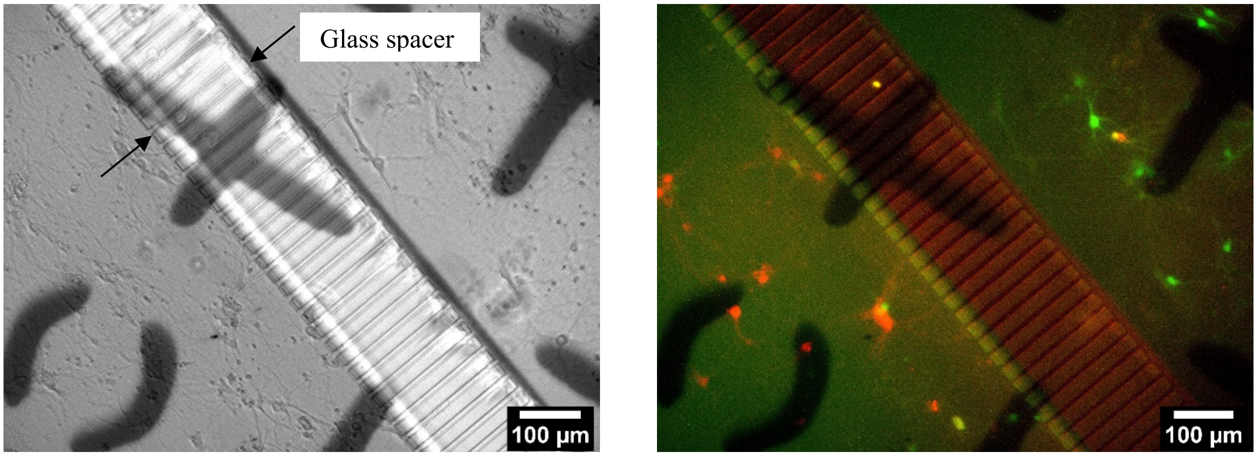

Supplementary Figure 4. Live imaging of 14 DIV postnatal hippocampal neurons growing on a structured and Poly-L-Lysine coated sapphire disc inside PDMS chamber. (A) 14 DIV neurons growing on patterned sapphire disc with extended neurites. The glass spacer which was aligned to cover microstructures is visible (at the tip of the arrows). (B) Two groups of postnatal hippocampal neurons labeled with mCherry and EGFP, respectively, which were growing in separated chambers. The relatively high background is due to slight misalignment of the sapphire and back-reflection of the glass spacer placed over the ridge structure.
